# Novel single-cell mtDNA sequencing technology reveals hidden mutations in oocytes, blastoids, and stem cells

**DOI:** 10.1093/lifemedi/lnad028

**Published:** 2023-07-25

**Authors:** Ismail M Shakir, Mo Li

**Affiliations:** Bioscience Program, Biological and Environmental Science and Engineering Division, King Abdullah University of Science and Technology, Thuwal 23955-6900, Kingdom of Saudi Arabia; Bioscience Program, Biological and Environmental Science and Engineering Division, King Abdullah University of Science and Technology, Thuwal 23955-6900, Kingdom of Saudi Arabia; Bioengineering Program, Biological and Environmental Science and Engineering Division, King Abdullah University of Science and Technology, Thuwal 23955-6900, Kingdom of Saudi Arabia

Mitochondria are small double-membraned organelles that are essential for cellular metabolism, reactive oxygen species (ROS) production, apoptosis, and calcium homeostasis. Mitochondria possess their own circular genome (mtDNA) of about 16.5 kb that encodes proteins and noncoding RNAs necessary for mitochondrial function. Although mitochondrial diseases can affect any organ, there is an obvious preference for organs with high energy demand such as the brain, skeletal muscle, and heart. mtDNA replicates independent of the cell cycle with a high turnover rate, making its genetic material prone to mutations. A typical cell contains a heterogenous mix of mutant and wild-type mtDNA, resulting in a certain heteroplasmy level that varies even between cells of the same tissue. mtDNA mutations that arise in oocytes become a basis of maternally inherited disease and can be inherited down the maternal lineage with multiple affected offspring. Conversely, low-frequency mtDNA mutations that arise in nondividing somatic cells of elderly, but otherwise healthy individuals can accumulate over time, causing a biochemical defect and contribute to aging.

Although next-generation sequencing (NGS) has been used to study mtDNA mutations, major challenges remain. First, conventional NGS-based methods rely on ensemble short-read sequencing of many cells, thereby averaging out the cell-to-cell heterogeneity and leading to inaccurate quantitation of heteroplasmy. Second, such averaging masks low-frequency mtDNA mutations and precludes the study of their functional significance in complex diseases and aging. Third, nuclear-mitochondrial DNA segments (NUMTs) when co-amplified in conventional NGS are challenging to remove using *in silico* approaches, especially when using short-read sequencing because of the presence of polymorphic NUMTs. Fourth, our understanding of the genetics behind maternally inherited mitochondrial diseases has been limited thus far because of the lack of methods for high-throughput analysis of the whole mutational landscape of single-mtDNA molecules in single oocytes. Fifth, mtDNA editing technologies are still in the early stages of development, and many technical challenges and safety concerns need to be addressed before they can be applied in clinical settings.

Two recent studies by Bi et al. [1, 2] present a novel sequencing technique—iMiGseq—that sufficiently overcomes current challenges in mtDNA sequencing and highlight the capability of the technique in understanding maternally inherited mitochondrial diseases through the study of oocytes, early development through human blastoid models, the role of mtDNA mutations in aging and cancers, and evaluation of the safety of two mtDNA editing strategies.

iMiGseq, short for individual Mitochondrial Genome sequencing, is based on a previously developed long-read individual molecule sequencing strategy technique by the same group [3]. iMiGseq uses a simple and fast procedure to barcode each mtDNA in single cells with a unique molecular identifier (UMI). UMI-labeled mtDNA are further amplified by high-fidelity long-range polymerase chain reaction (PCR) and sequenced on the long-read nanopore sequencing platform commercialized by Oxford Nanopore Technologies to obtain full-length mtDNA, quantitate variant frequency and determine the complete haplotype of individual mtDNA.

iMiGseq demonstrated its ability to accurately characterize mtDNA heterogeneity, thus allowing for a quantitative understanding of mtDNA genetics in single oocytes. iMiGseq identified high-confidence UMI groups and single nucleotide variants (SNVs) therein in oocytes of the NSG and C57/BL6 strains of mice. Most SNVs had a heteroplasmy level below 1%, which is the detection limit of conventional NGS technologies. iMiGseq showed excellent accuracy, with no false SNVs detected at a polymorphic position between the two strains. iMiGseq further uncovered significant differences in the frequency of the same variant among three oocytes from the same mouse. These findings suggest that the frequency of ultra-rare detrimental variants can increase during oogenesis, highlighting the need for ultra-sensitive techniques like iMiGseq. The authors constructed phylogenetic trees based on mtDNA haplotypes, which provided evidence of sequential acquisition of *de novo* mutations in individual mtDNA. The long reads generated from iMiGseq allowed for the phasing of novel variants with tissue-specific homoplasmic SNVs and confidently avoided NUMTs.

Bi et al. also extended their work to humans and presented the first single-mtDNA analysis of heteroplasmy profile in single healthy human oocytes. iMiGseq identified a hitherto unappreciated prevalence of rare pathogenic heteroplasmic variants well below the detection limit of conventional NGS methods. Moreover, their results revealed that the frequency of rare heteroplasmic variants can fluctuate significantly among oocytes. Quantitative genetic linkage analysis revealed significant shifts in variant frequency and clonal expansions of large SVs during oogenesis in single-donor oocytes. iMiGseq of a single human blastoid [4], a stem cell-based model of human blastocyst, reported stable heteroplasmy levels during early lineage differentiation of naïve pluripotent stem cells. These experiments presented a strategy to follow mtDNA dynamics in normal and diseased human blastoid models.

iMiGseq also proved to facilitate the genetic diagnosis of mitochondrial disease. When applied to induced pluripotent stem cells (iPSCs) from a patient diagnosed with NARP (neuropathy, ataxia, retinitis pigmentosa) and Leigh syndrome, iMiGseq not only correctly determined the frequency of the mutation but also identified five SNVs missed by whole genome sequencing. Furthermore, iMiGseq generated haplotype-resolved single mtDNA reads and identified a large deletion in the D-loop region.

Characterizing the outcome of mitochondrial genome editing tools is paramount to determining their safety for clinical use. However, current methods of evaluating mitochondrial genome editing outcomes by conventional deep NGS fail to detect complex SVs and variant allele frequency changes of low-frequency variants (on- and off-target variants). Using Mitochondrial encephalomyopathy, lactic acidosis, and stroke-like episodes (MELAS) patient iPSCs, iMiGseq revealed that mitoTALEN corrected the MELAS mutation, but, surprisingly, caused an unintended near homoplasmic shift of a new mutation linked with the wild-type allele following the restoration of mtDNA copy number. In addition, iMiGseq detected a pathogenic SNV with 1.07% frequency while parallel deep NGS failed to report it. Conversely, iMiGseq confirmed the DddA-derived cytosine base editor’s [5] preference for editing specific bases and showed that the heteroplasmic levels of SNVs remained stable after editing with no unintended mutations above the background.

**Figure 1.**
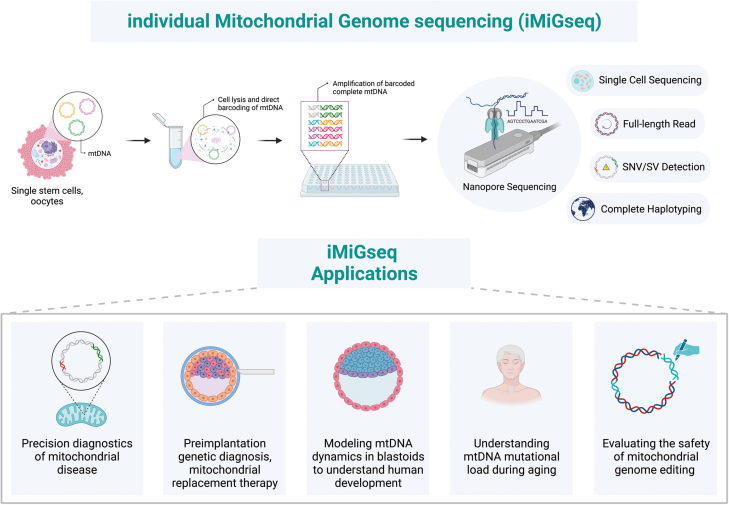
iMiGseq and its applications. iMiGseq is a novel mtDNA sequencing technique that exploits the long-read sequencing ability of nanopore sequencing and provides an unbiased, high-throughput, base-resolution analysis of the mutational landscape of individual full-length mtDNA in single cells. iMiGseq holds the potential to catalyze advances in precision diagnostics of mitochondrial diseases, improve the success rates of conventional ART such as PGD and support emerging technologies such as MRT. iMiGseq will help understand mtDNA dynamics during early human development modeled through blastoids. It can provide researchers with a deep dive into the role of mitochondrial dysfunction in aging and age-related degenerative diseases by uncovering low-frequency mutations that occur in somatic cell types. iMiGseq also presents itself as a valuable tool to evaluate the safety of existing and future mitochondrial genome editing tools. This figure was created with BioRender.com.

iMiGseq holds the potential to unlock our understanding of several areas including mitochondrial disease diagnosis, assisted reproductive technologies (ART), and aging-associated complex diseases. Epidemiological studies have estimated that ~1 in 4,300 individuals is affected by primary mtDNA diseases. Accurate diagnosis of most mitochondrial-related diseases is particularly challenging because of the multi-system clinical presentation patients present with. iMiGseq as a sensitive, high-throughput sequencing technique can facilitate mitochondrial disease diagnosis. Owing to its ability of detecting heteroplasmy levels below 1%, iMiGseq can uncover novel mutations and expand our fundamental and clinical understanding of mitochondrial diseases (Fig. 1). Correlating accurate heteroplasmy levels with clinical presentations can help physicians determine the threshold level for disease expression and severity. Moreover, iMiGseq-generated heteroplasmy levels can be incorporated into the existing Mitochondrial Disease Criteria scoring system to determine the likelihood of mitochondrial disease in suspected patients and to pinpoint a genetic diagnosis. The ultra-sensitive sequencing data can be used to construct pedigree charts for affected families and calculate carrier probabilities with better sensitivity rates, thereby assisting genetic counselors in providing effective counseling.

Screening for mitochondrial mutations with iMiGseq is also pertinent to cancer diagnosis. mtDNA mutations are common in all tumors and directly impact metabolic homeostasis. Recent large-scale sequencing projects have emphasized the prevalence of mtDNA mutations in tumors and their possible role in oncogenesis. As such, screening for mtDNA mutations (including cell-free mtDNA) can offer potential biomarkers for cancer diagnosis and prognosis. By constructing a patient-specific mtDNA mutation landscape, iMiGseq could facilitate the identification of biomarkers for better cancer diagnosis and prognosis, and monitoring of chemotherapy.

With regard to ART (Fig. 1), iMiGseq can be used to assess mitochondria from blastomeres during preimplantation genetic diagnosis (PGD) to ensure that the baby will be free of mitochondrial disease. iMiGseq could also be applicable to mitochondrial replacement therapy (MRT), especially in women with high levels of heteroplasmy or a high frequency of homoplasmic variants. MRT is often employed when PGD cannot benefit mothers who harbor homoplasmic mtDNA mutation. iMiGseq can be a powerful tool for screening for harmful mutations in a small fraction of the mitochondria to be transferred and can provide definitive evidence for the quality of the donated mtDNA.

iMiGseq can enhance our understanding of the complex relationship between aging and age-related diseases by providing detailed atlases of mtDNA mutations. iMiGseq can uncover mtDNA mutations that are correlated with age-related diseases and hence potential contributors to the aging process (Fig. 1). Moreover, since mitochondrial dysfunction is a hallmark of aging, by examining the mitochondrial genome closely using iMiGseq, we can gain insights into the mechanisms underlying mitochondrial dysfunction and identify potential targets for therapeutic interventions. Furthermore, there is emerging evidence suggesting the role of nuclear-mitochondrial signaling in regulating mitochondrial function and aging. iMiGseq can help uncover the extent and nature of these interactions, offering a more comprehensive understanding of aging.

While mtDNA sequencing has already demonstrated significant clinical value, ongoing developments such as iMiGseq that offer single-molecule, long-read sequencing with low input requirement, higher sensitivity, and better SV-resolving power, especially within key single cells such as oocytes, promise a holistic view of the mitochondrial genome that will undoubtedly improve patient care and advance precision diagnostics.
